# Regulatory T cells protect from autoimmune arthritis during pregnancy

**DOI:** 10.1016/j.jaut.2011.09.007

**Published:** 2012-05

**Authors:** Alba Munoz-Suano, Marinos Kallikourdis, Milka Sarris, Alexander G. Betz

**Affiliations:** Medical Research Council, Laboratory of Molecular Biology, Hills Road, Cambridge CB2 0QH, United Kingdom

**Keywords:** Rheumatoid arthritis, Pregnancy, Regulatory T cells, Mouse model, RA, rheumatoid arthritis, T_R_, regulatory T cells, CIA, collagen-induced arthritis, PAMPs, pathogen-associated molecular patterns

## Abstract

Pregnancy frequently has a beneficial effect on the autoimmune disease Rheumatoid Arthritis, ranging from improvement in clinical symptoms to complete remission. Despite decades of study, a mechanistic explanation remains elusive. Here, we demonstrate that an analogous pregnancy-induced remission can be observed in a mouse model of arthritis. We demonstrate that during pregnancy mice are protected from collagen-induced arthritis, but are still capable of launching normal immune responses to influenza infections. We examine the role of regulatory T (T_R_) cells in this beneficial effect. T_R_ cells are essential for many aspects of immune tolerance, including the suppression of autoimmune responses. Remarkably, transfer of regulatory T cells from pregnant ‘protected’ mice was sufficient to confer protection to non-pregnant mice. These results suggest that regulatory T cells are responsible for the pregnancy-induced amelioration of arthritis.

## Highlights

► Pregnancy-induced protection from RA is not due to systemic immunosuppression. ► The effect can be studied in Collagen-Induced Arthritis, a mouse model of human RA. ► Protection can be transferred from pregnant-protected to control mice using T_R_ cells. ► The pregnancy-induced suppression of RA by T_R_ cells is antigen-specific.

## 1 Introduction

Rheumatoid Arthritis is an autoimmune disease predominantly affecting post-menopausal women, but that can also affect women of childbearing age [1]. As a consequence, clinicians are faced with difficult choices regarding the selection of an optimal therapeutic regime that deals with the symptoms of the disease without negatively affecting the pregnancy, as some of the therapeutic regimes for RA are unsafe for use during pregnancy [2]. Further, women with RA have an increased risk of adverse pregnancy outcomes [3–5]. A better understanding of the mechanism driving the pregnancy-associated changes in RA will provide us with valuable information to help resolve this problem. In addition, it will provide useful clues regarding the pathogenesis of RA in general, thus opening the way to the development of novel treatments [6,7].

Frequently, disease activity in patients with RA decreases spontaneously during pregnancy ranging from an improvement in clinical symptoms to complete remission. However, this effect is transient and the disease relapses shortly after delivery [8]. This pregnancy-induced amelioration of RA symptoms was first described by Hench in 1938 [9] and was a crucial hint towards the identification of corticosteroids as immunosuppressive drugs for use in treating autoimmunity (Table 1) [6]. Since then, a large number of retrospective and prospective studies on RA patients have confirmed that an improvement in disease activity occurs during pregnancy in half to three quarters of patients [8,10–12]. It is noteworthy that the higher efficacy of more recently developed therapeutic regimes is thought to lead to lower levels of RA activity in patients prior to pregnancy, thus partially “masking” the beneficial effect of pregnancy in more recent reports [7]. Despite decades of study, a mechanistic explanation for the pregnancy-induced remission and post-partum relapse of RA remains elusive [12].

Here, we examine the role of CD25^+^ regulatory T (T_R_) cells in this beneficial effect. T_R_ cells are a naturally occurring subpopulation of T cells that are essential for many aspects of immune tolerance, including the suppression of autoimmune responses [13]. During pregnancy they protect the fetus from rejection by the maternal immune system in both mice [14] and humans [15–17] (Table 2). The accumulation of antigen-experienced T_R_ cells in the uterus [18] suggests that the suppression of the anti-fetal immune response occurs in a localized and antigen-specific fashion. Further support for an antigen-specific action of T_R_ cells comes from studies examining the immune response to the minor transplantation antigen H-Y in the context of maternal–fetal tolerance [19]. A hint regarding an involvement of T_R_ cells in the amelioration of RA comes from the observation that their number inversely correlates with disease activity during pregnancy [20] (Table 1). However, experimental proof for a mechanistic involvement of T_R_ cells remained outstanding.

To examine whether T_R_ cells mediate the pregnancy-associated remission of arthritis, we studied the phenomenon in Collagen-Induced Arthritis (CIA), a mouse model of the disease. We found that pregnancy protects the mice from developing arthritis. Transfer of CD25^+^ cells from these pregnant-protected mice into non-pregnant recipients protected them from CIA. The fact that transfer of CD25^+^ cells from pregnant mice that were not exposed to CIA induction did not confer protection to the recipients suggests that the T_R_ cells act in an antigen-specific fashion.

## 2 Materials and methods

### 2.1 Animal care

All animal care was provided by expert animal technicians, in compliance with the relevant laws and institutional guidelines.

### 2.2 Influenza infections

C57BL/6 females and C57BL/6 females mated with BALB/c males were infected intra-nasally with 10^4^ PFU of HKx31(H3N2) virus [21] under iso-fluorane anesthesia on the first day of pregnancy (as determined by detection of a vaginal plug). On day 10 after infection antigen-specific cells were identified using PE and APC conjugated H-2Db/NP ASNENMETM pentamers (Proimmune) and anti-mouse CD8 FITC (eBioscience, clone 53–6.7) by FACS.

### 2.3 Collagen-induced arthritis

Female C57BL/6 mice received an intra-dermal injection of 100 μl of 100 μg chicken collagen type II (Sigma) in Complete Freund’s Adjuvant, on day 0 and day 21 and were monitored for clinical signs of CIA on a daily basis. The humane endpoint for this series of experiments was set when the mice reached a clinical score [22] of ≥8 out of 12. Some of the mice were set up for mating with BALB/c males from day 31–35 (one estrus cycle). All mice that reached a clinical score above 6 prior to the day of the set up of matings were excluded from the experiment, irrespective of whether they partook in matings or not.

### 2.4 Cell purifications

Cell suspensions of spleen, lymph nodes and uterus were prepared by gently forcing the tissues through 70 μm-pore cell strainers. Lymphocytes were isolated by Lympholyte (Cedarlane) gradient centrifugation according to manufacturer’s instructions, pooled and stained with anti-mouse CD25-PE antibody (clone 7D4, BD). After incubation with anti-PE beads (Miltenyi Biotec) the cells were isolated using MS columns (Miltenyi Biotec) according to manufacturers instructions and the purity assessed by FACS. Cells were re-suspended in PBS and intravenously injected into mice.

### 2.5 Adoptive transfer

**Fig. 1:**
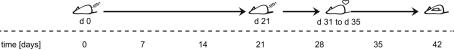
Timeline of CIA inductions and matings. CIA was induced by intra-dermal injection of C57BL/6 mice with chicken collagen type II in Complete Freund’s Adjuvant (syringe) on day 0 and day 21. The mice were set up to mate with allogeneic BALB/c males from day 31 to day 35 (hearts).

The experimental designs are outlined in Figs. 1 and 3. CIA-induced C57BL/6 females received an adoptive transfer of CD25^+^ cells 31 days after the start of CIA induction. The cells used were prepared from C57BL/6 females that were treated to induce CIA, mated and sacrificed on day 9.5–11.5 of gestation (pregnant-protected), or did not receive any CIA induction but were mated and sacrificed at the same time (pregnant), or were neither treated to induce CIA nor mated (non-pregnant). For 1:1 transfers all CD25^+^ cells from one donor were adoptively transferred into one recipient, irrespective of the cell number. As our emphasis was to minimize loss of T_R_ cells during purification, we followed a protocol optimized for high yield of CD25^+^ cells, typically achieving >50% purity. None of the pregnant-protected mice used as donors showed any signs of arthritis (in all cases the clinical score was <3).

### 2.6 Statistical analyses

Statistical analyses were performed using GraphPad Prism and Excel as appropriate.

## 3 Results

**Fig. 2:**
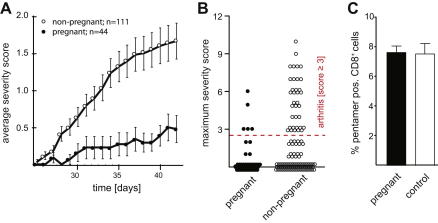
Pregnancy protects from CIA. (A) Time course (days after CIA induction) of the average severity (*P* = 0.0002, two-tailed Wilcoxon signed rank test) or (B) the maximum severity reached (*P* = 0.0136, two-tailed Fisher’s exact test for score <3 versus score ≥3). (C) Percentage of NP-pentamer^+^ CD8^+^ cells in the spleen 10d after intra-nasal HKx31 influenza infection on the first day of pregnancy (non-pregnant: *n* = 9; pregnant: *n* = 5; *P* = 1, two-tailed unpaired *t*-test). Error-bars represent the standard error of the mean.

CIA in mice resembles the pathology of RA both in terms of histopathology and serological biomarkers [22,23]. To induce arthritis in C57BL/6 mice we injected them with chicken Collagen Type II in Complete Freund’s Adjuvant intra-dermally on day 0 and day 21. Some of the mice were mated allogeneically with BALB/c males on days 31–35 (Fig. 1). We compared the course of CIA in non-pregnant (*n* = 111) and pregnant (*n* = 44) mice and found that pregnancy protected the mice from the disease (incidence of 32% vs. 11%; Table 3). This is reflected in both the average clinical score over time (*P* = 0.0002, two-tailed Wilcoxon signed rank test; Fig. 2A) and the maximum clinical score reached (Fig. 2B).

To verify that this is not due to a pregnancy-induced systemic immunosuppression, we compared the response to intra-nasal influenza HKx/31(H3N2) infection in pregnant (*n* = 5) and non-pregnant mice (*n* = 9). We found that pregnancy had no effect on the expansion of CD8^+^ cells specific for the H–2D^b^/nucleoprotein (NP) peptide complex (non-pregnant vs. pregnant; 7.44 ± 0.65 vs. 7.48 ± 0.51; *P* = 1, two-tailed unpaired *t*-test; Fig. 2C and Table 4). This demonstrates that pregnant mice are capable of launching normal immune responses against this pathogen. Thus, the protection from arthritis cannot be due to a pregnancy-induced systemic immune suppression.

**Fig. 3:**
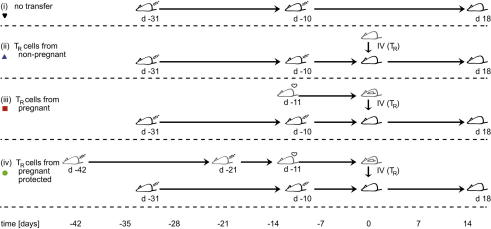
Timeline of CIA inductions and adoptive transfer of T_R_ cells. Donors are shown in grey and recipients shown in black. All recipients were CIA-induced and split into 4 groups. (i) received no T_R_ cell graft (no transfer), (ii) received T_R_ cells isolated from non-pregnant donors (non-pregnant), (iii) received T_R_ cells isolated from pregnant donors (pregnant) and (iv) received T_R_ cells isolated from donors that were protected from the disease by pregnancy despite of CIA induction (pregnant-protected). The exact timing of the various inductions (syringe), matings (heart) and adoptive transfers (IV (T_R_)) are shown on the timeline. Adoptive transfers were performed on day 0.

**Fig. 4:**
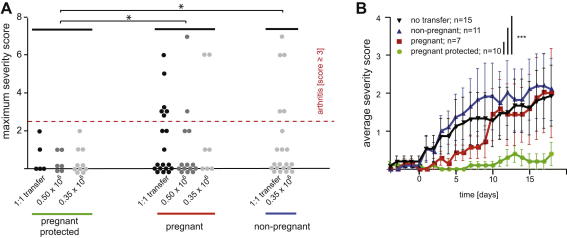
Regulatory T cells mediate pregnancy-induced protection from arthritis. The effect of adoptive transfer of CD25^+^ cells prepared from either non-pregnant, pregnant or ‘CIA-induced’ pregnant (pregnant-protected) into non-pregnant mice, in which CIA had been induced 31 days earlier. (A) The indicated number of cells was transferred and the maximum severity score reached is shown. For the calculation of the statistical significance the animals were grouped irrespective of the number of cells (3.5 × 10^5^–12 × 10^5^). *P < 0.05 (two-tailed Fischer’s exact test for score <3 versus score ≥3; Pregnant-protected versus pregnant *P* = 0.0234; pregnant-protected versus non-pregnant *P* = 0.0478). (B) 3.5 × 10^5^ cells were transferred and a time course (days after transfer) of the average severity is shown (two-tailed Wilcoxon signed rank test; *** indicates *P* < 0.001; pregnant-protected versus pregnant *P* = 0.0005, pregnant-protected versus non-pregnant *P* = 0.0003, pregnant-protected versus no transfer *P* < 0.0001). Error-bars represent the standard error of the mean.

To investigate whether the protection from CIA during pregnancy can be attributed to the action of T_R_ cells, we ‘substituted’ pregnancy with adoptive transfer of CD25^+^ cells (Fig. 3). Non-pregnant mice, in which CIA had been induced, received CD25^+^ cells sourced from either non-pregnant control mice (non-pregnant; *n* = 21), untreated pregnant mice (pregnant; *n* = 19), or mice that were protected from the disease by pregnancy despite CIA induction (pregnant-protected; *n* = 5). Each recipient mouse received all CD25^+^ cells obtained from a donor mouse in a one-to-one fashion. Whilst none of the mice receiving CD25^+^ cells from pregnant-protected donors developed arthritis, 24% (5 out of 21) of recipients of cells from non-pregnant donors and 32% (6 out of 19) of the recipients of cells from pregnant untreated donors developed arthritis (Fig. 4A; 1:1 transfer).

The number of CD4^+^CD25^+^ cells significantly increases during pregnancy from 0.35 to 0.5 × 10^6^ cells in non-pregnant mice to approx. 1.5 × 10^6^ cells in pregnant mice [14]. Therefore, we titrated the number of cells transferred to match the numbers that can be obtained from non-pregnant donors. Transfer of 0.35 × 10^6^ CD25^+^ cells from control mice had no effect on the outcome of CIA in the recipients (no transfer vs. non-pregnant; Table 5 and Fig. 4B). Whilst transfer of the same number of CD25^+^ cells from pregnant mice appeared to cause a slight delay in the onset of clinical signs (pregnant, Fig. 4B), the outcome *per se* was not affected (no transfer vs pregnant; Table 5). In contrast, none of the recipients of CD25^+^ cells from pregnant-protected mice developed any signs of arthritis (pregnant-protected, Fig. 4B and Table 5).

In summary, we observed a significant protection from CIA (pregnant-protected, *P* < 0.05, two-tailed Fischer’s exact test; Fig. 4A) irrespective of the number of cells transferred and conclude that T_R_ cells mediate the pregnancy-associated protection from CIA. The fact that T_R_ cells from pregnant mice that did not undergo CIA induction did not protect from arthritis (non-pregnant vs. pregnant; Table 6 and Fig. 4A) shows that the pregnancy by itself is insufficient to protect from arthritis. Rather, our data suggest that this protective effect requires prior exposure of the T_R_ cells to arthritis-related antigens in the context of pregnancy (non-pregnant vs. pregnant-protected; Table 6 and Fig. 4A).

## 4 Discussion

Since the first description of the pregnancy-induced amelioration of RA symptoms, numerous studies have attempted to elucidate the underlying mechanism (Table 1). Pioneering work by Whyte and co-workers used a model of CIA in DBA mice to examine both the amelioration of arthritis during pregnancy and the post-partum relapse of the disease [24]. Their results suggested that prolactin [25] and oestradiol [26] have opposite effects on the post-partum course of the disease. Yet, due to the lack of precise temporal correlation with disease activity, doubts were expressed on the role of hormones in this process [10]. A better temporal correlation with disease activity was observed for the percentage of IgG-associated agalactosyl N-linked oligosaccharides, which decreases during the amelioration of arthritis [27]. However, this could not be explained by a pregnancy-induced clearance of the agalactosyl IgG by mannose-binding lectin [28].

A further line of investigation centered on the observation that allogeneically mated B10.RIII females were more protected from CIA than syngeneically mated females [29]. This has been attributed to both changes in the ratio of T cell populations [30] and changes in cytokine levels [31]. In humans, the extent of disparity in HLA-DP and HLA-DQ MHC Class II molecules between the mother and the fetus was found to correlate with remission from arthritis during pregnancy [32–34], though a later study on inflammatory polyarthritis did not find such a correlation [35].

Several lines of evidence have suggested that T_R_ cells have a role in the regulation of arthritis [36]. T_R_ cells in RA patients show functional defects [37] and depletion of T_R_ cells in mice leads to increased disease severity [38]. Here, we demonstrate that T_R_ cells from pregnant-protected mice are sufficient to confer protection from CIA when transferred into non-pregnant mice. This strongly suggests that T_R_ cells are responsible for the pregnancy-induced amelioration of RA.

Prior to pregnancy, exposure of the mother to paternal transplantation antigens induces a rigorous immune response against the graft [39]. In the context of pregnancy, this response is suppressed to prevent a rejection of the fetus [14]. It appears that some autoimmune responses such as rheumatoid arthritis and multiple sclerosis [40] are also re-assessed during pregnancy, resulting in a temporary amelioration of these diseases.

Autoimmune responses could potentially be suppressed in an antigen-specific fashion or by bystander effects. The accumulation of antigen-experienced T_R_ cells in the gravid uterus [18] suggests that the suppression of the anti-fetal immune response occurs in a localized and antigen-specific fashion (see also Table 2). Similarly, in the case of autoimmune diabetes, the data points to a highly antigen-specific involvement of T_R_ cells [41]. Further support for an antigen-specific action of T_R_ cell comes from our endeavours to find a cell-mediated therapy for arthritis. Genetically engineered inducible Foxp3 (iFoxp3) can be used to confer T_R_ cell phenotype to T_H_ cells [42]. This can be used to stop CIA using iFoxp3-transduced, polyclonal T cell autografts. We found that this approach only worked if the iFoxp3-transduced T_H_ cells were exposed to arthritis antigens prior to switching on Foxp3 [42]. If iFoxp3 was switched on prior to exposure to arthritis antigens, the course of the disease was not affected. All these findings point towards an antigen-specific suppression by T_R_ cells. The data presented here provide evidence that the amelioration of arthritis during pregnancy is also antigen-specific. Only T_R_ cells isolated from ‘pregnant-protected’ mice conferred arthritis protection to non-pregnant mice. T_R_ cells from pregnant mice that had not been exposed to arthritis-related antigens could not confer protection.

Some mechanistic insight comes from the observation that pregnancy is accompanied by a shift from T_H_1 to T_H_2 type responses. It has been suggested that this in itself might lead to a diminution of the underlying immune response driving RA [43,44]. Pregnant women with RA display a reduction in the capacity of their peripheral blood mononuclear cells to produce the T_H_1 cytokines IL-12 and IFNγ [45]. This hypothesis could explain why some autoimmune diseases such as SLE can exhibit flares during pregnancy, presumably due to a T_H_2 bias of the underlying immune response [46]. It remains to be seen whether the T_H_1/T_H_2 shift during pregnancy acts in parallel to the action of T_R_ cells or whether the change in bias is actually mediated by the T_R_ cells. It is noteworthy that in contrast to the essential requirement for T_R_ cells, a change in the T_H_1/T_H_2 bias is not fundamental to maternal–fetal tolerance, as mice deficient in T_H_2 cytokines can become allogeneically pregnant [47].

We propose that the amelioration of arthritis is a collateral consequence of the immune system’s reassessment of all responses coinciding with pregnancy. By making context-dependent decisions, the immune system can suppress immune responses directed against the fetus whilst remaining vigilant towards pathogens, such as influenza, that are recognized to be a danger to the mother. The finding that pathogen-associated molecular patterns (PAMPs) under certain, specific conditions can block T_R_-mediated suppression [48] offers a hint to as to how the immune system might interpret the context. The absence of exogenous ‘danger’ signals in ongoing autoimmune responses might be sufficient for the immune system during pregnancy to reassess and suppress them. One might speculate that the transient nature of the pregnancy-associated suppression is of evolutionary advantage, as a more permanent induction of tolerance would be prone to be exploited by pathogens. Indeed, certain pathogens, such as Listeria and Salmonella, appear to be able to take advantage of the pregnancy-induced tolerance mechanisms, as these infections are exacerbated by pregnancy [49]. The exact mechanism by which immune responses coinciding with pregnancy are re-interpreted by the immune system warrants further investigation.

## Disclosures

The authors declare that they have no competing financial interests.

## Figures and Tables

**Table 1:** Mechanism implicated in the pregnancy-associated amelioration of arthritis.

Human studies	Mouse studies	Comment
The immune-modulatory action of corticosteroids was suspected to improve RA during pregnancy [6]		This has subsequently been shown not to be relevant in this context [6]
T_H2_ shift during pregnancy might redirect the immune response [43–45]		There is no essential role for T_H2_-associated cytokines in maternal–fetal tolerance [47]
	Prolactin [25] is associated with the post-partum relapse of symptoms, whilst oestrogen appears to have the opposite effect [26]	The kinetics of hormonal changes after delivery does not match that of the relapse of symptoms [10]
IgG-associated agalactosyl falls during pregnancy in patients and is inversely correlated to disease severity [27]		The mechanism of this observation remains to be elucidated [28]
MHC disparity between mother and fetus is correlated to the amelioration of RA during pregnancy [32,33]	Allogeneic pregnancy is associated with increased amelioration of arthritis [30,31]	Extensive data, though some is conflicting [35]
Correlation between the number of T_R_ cells and the pregnancy-induced amelioration of RA [20]		

**Table 2:** The role of T_R_ cells in pregnancy and arthritis.

Human studies	Mouse studies	Comments
T_R_ cells are associated with maternal–fetal tolerance [15–17]	T_R_ cells are necessary for maternal–fetal tolerance [14]	
	T_R_ cell-mediated maternal–fetal tolerance is antigen-specific [18,19]	
T_R_ cells defective in RA patients [37]		
	Ablation/depletion of T_R_ cells exacerbates arthritis [36,38,50]	These studies indicate that T_R_ cells are involved in the regulation of RA associated immune responses.
	Adoptive transfer of polyclonal pre-stimulated T_R_ cells can reduce signs of arthritis [51,52]	Adoptive transfer of non-activated polyclonal T_R_ cells has no effect on arthritis [53]
	iFoxp3-transduced cells can be induced to assume T_R_ cell phenotype and prevent arthritis in an antigen-specific fashion [42]	This ‘Trojan horse’ approach circumvents the requirement of pre-activation of the cells and makes the suppression antigen-specific
	Danger signals break T_R_ cell-mediated tolerance [48]	Some pathogens can exploit the T_R_ cell-mediated pregnancy-induced reassessment of immune status [49]

**Table 3:** Amelioration of CIA during pregnancy.

Status of animal	No. of mice	Incidence
Non-pregnant	111	35/111 (32%)
Pregnant	44	5/44 (11%)

Results show the total number of individual mice in 7 independent experiments.

**Table 4:** Response to influenza infection.

Status of animal	No. of mice	Pentamer^+^CD8^+^ cells [%]
Non-pregnant	9	7.44 ± 0.65 (SEM)
Pregnant	5	7.48 ± 0.51 (SEM)

Results are represented as percentage of antigen-specific CD8^+^ cells ± SEM.

**Table 5:** Clinical features of CIA after transfer of 0.35 × 10^6^ CD25^+^ cells.

Type of donor	No. of mice	Incidence
no transfer	15	3/15 (20%)
non-pregnant	11	4/11 (36%)
pregnant	7	2/7 (29%)
pregnant-protected	10	0/10 (0%)

Results show the total number of individual mice in 3 independent experiments.

**Table 6:** Clinical features of CIA after transfer of CD25^+^ cells, irrespective of the number of cells transferred.

Type of donor	No. of mice	Incidence
non-pregnant	21	5/21 (24%)
pregnant	42	10/42 (24%)
pregnant-protected	21	0/21 (0%)

Results show the total number of individual mice in 6 independent experiments.
